# Removal of ^241^Am from Aqueous Solutions by Adsorption on Sponge Gourd Biochar

**DOI:** 10.3390/molecules28062552

**Published:** 2023-03-10

**Authors:** Maria Philippou, Ioannis Pashalidis, Dimitrios Kalderis

**Affiliations:** 1 Department of Chemistry, University of Cyprus, P.O. Box 20537, Nicosia 1678, Cyprus; 2 Laboratory of Environmental Technologies and Applications, Department of Electronic Engineering, Hellenic Mediterranean University, 73100 Chania, Greece

**Keywords:** americium, radioactivity, biochar, environmental waters, adsorption

## Abstract

*Luffa cylindrica* biomass was converted to biochar and the removal of ^241^Am by pristine and oxidized biochar fibers was investigated in laboratory and environmental water samples. This species has the added advantage of a unique microsponge structure that is beneficial for the production of porous adsorbents. The main purpose of this study was to valorize this biomass to produce an efficient adsorbent and investigate its performance in radionuclide-contaminated waters. Following the preparation of Am^3+^ solutions at a concentration of 10^−12^ mol/L, the adsorption efficiency (*K*_d_) was determined as a function of pH, adsorbent mass, ionic strength, temperature, and type of aqueous solution by batch experiments. At the optimum adsorbent dose of 0.1 g and pH value of 4, a log_10_*K*_d_ value of 4.2 was achieved by the oxidized biochar sample. The effect of temperature and ionic strength indicated that adsorption is an endothermic and entropy-driven process (Δ*H*° = −512 kJ mol^−1^ and Δ*S*° = −1.2 J K^−1^ mol^−1^) leading to the formation of inner-sphere complexes. The adsorption kinetics were relatively slow (24 h equilibrium time) due to the slow diffusion of the radionuclide to the biochar surface and fitted well to the pseudo-first-order kinetic model. Oxidized biochar performed better compared to the unmodified sample and overall appears to be an efficient adsorbent for the treatment of ^241^Am-contaminated waters, even at ultra-trace concentrations.

## 1. Introduction

Americium is a man-made, radioactive metal, and its most common isotopes are ^241^Am and ^243^Am. In small amounts, americium is present in uranium minerals due to nuclear reactions that may occasionally occur. Most americium is produced in nuclear reactors when neutrons are captured by uranium or plutonium, and about 100 g of americium is contained in one ton of spent nuclear fuel. No natural sources of americium exist, therefore, its presence in the environment is mainly due to nuclear weapons testing or accidental releases from nuclear power plants [1,2]. The americium isotope ^241^Am (t_1/2_ = 432.2 y) can be easily produced in pure form and therefore has several applications. It is used in smoke detectors, lightning rods, and portable sources of alpha and gamma rays, which have found application in a number of medical and industrial uses. Particularly, the 60 keV gamma ray emission of ^241^Am is used in radiography and X-ray fluorescence spectroscopy for material analysis and quality control [1,2]. The appropriate handling and disposal of such sources is critical because americium released in the environment may cause health effects to living organisms including humans. After uptake, americium is rapidly transported to the bones where it can be stored for a long period of time. There, the radionuclide decays slowly, emitting alpha-particles and gamma rays, which can alter genetic material and cause bone cancer [1,2]. Hence, it is obligatory to remove americium from contaminated environments including natural waters and wastewaters.

Although several oxidation states are known, ranging from +2 to +7, ^241^Am in solutions exists predominantly in the trivalent oxidation state (Am^3+^). The speciation of Am^3+^ in aqueous solutions is strongly dependent on the solution pH. Under ambient conditions and acidic pH values, the Am^3+^ cation is the predominant species, whereas at near neutral pH values, Am^3+^, Am(OH)_2_^+^, and Am(CO_3_)^+^ are the dominating species. At alkaline pH values, the negatively charged carbonate complexes of Am^3+^ (e.g., Am(CO_3_)_2_^−^) are the predominant species, which generally stabilize the radionuclide, resulting in lower removal efficiencies [3,4,5,6,7].

A wide spectrum of physical, chemical, and biological processes has been developed and applied for the removal of radionuclides from the nuclear process and contaminated wastewaters including membrane and osmosis technologies, sorption and ion exchange, chemical precipitation, electroremediation, and bioremediation [8]. Adsorption technologies are of particular interest since effective adsorbents such as metal organic frameworks [9], aerogels [10,11,12], silica-based materials [13], polymers [14,15], biomasses [16,17,18,19], and carbon-based composites [20,21,22,23,24,25] can be prepared easily and at low-cost. One such material is biochar, prepared by the pyrolysis of residual dry biomass and agricultural waste. The properties of biochar depend on the initial feedstock properties and pyrolysis conditions. As a result, multi-functional, engineered biochars can be produced to address the requirement of a specific application.

In recent years, the use of modified biochar fibers has been extensively investigated, indicating that these materials could substitute activated carbon for the adsorption of radionuclides such as uranium [26,27,28]. Additionally, KOH-activated biochar has shown that it can achieve more than 80% removal of ^137^Cs and 47% of ^90^Sr, whereas the high removal capacity was attributed to the increased surface area and pore volume of the adsorbent [29]. A high (>99%) removal of ^90^Sr was also achieved by peanut shell biochar prepared at 400 °C, whereas the presence of OH and C–H on the biochar surface were responsible for high adsorptive capacity [30]. The applicability of biochar has also been demonstrated in soils contaminated with ^238^U and ^232^Th, where the phytoaccumulation of the radionuclides on cabbage was minimized [31]. However, another study that applied biochar in soil to control the activity of ^137^Cs, ^40^K, ^226^Ra, ^214^Bi, ^214^Pb, and ^228^Ac achieved limited results [32].

The removal of ^241^Am from environmental bodies has not received adequate attention and the published literature is limited. Zhang et al. (2014) prepared phosphonic acid-functionalized silicas with different mesoporous morphologies and surface areas in the range of 521–769 m^2^/g, which were tested in the adsorption of Am^3+^ [33]. The authors achieved the highest adsorption capacities (48–57 mg/g) at pH = 3.3 for all silicas. Silica was also the substrate in a different work, where the authors deposited isobutyl-2,6-bis(5,6-dibutyl-1,2,4-triazin-3-yl) pyridine on the pores of SiO_2_–P particles to prepare an adsorbent for ^241^Am and rare earth removal [34]. The composite adsorbent exhibited good adsorption selectivity for ^241^Am over several rare earths in acidic conditions, whereas 80% of ^241^Am was removed after only 0.5 h. A similar efficiency and overall behavior were exhibited by an adsorbent prepared by depositing bi(2-ethylhexyl) phosphoric acid on SiO_2_–P [35]. More recently, a porous acrylic polymer was impregnated with surfactants and applied to the removal of ^241^Am, achieving adsorption capacities in the range of 10–17 mg/g [36].

Given the lack of information and the hazardous nature of ^241^Am, the rationale of this work was to determine whether ^241^Am-contaminated waters can be treated with a carbonaceous adsorbent prepared from waste biomass. For this purpose, the subtropical plant of *Luffa cylindrica* (vegetable sponge gourd) was selected as the biochar precursor. Compared to other biomasses, this plant has the added advantage of a microsponge structure with 200–500 microcellular fibers, which renders it a promising adsorbent material [37]. Therefore, the specific objectives were to (a) investigate the removal of ^241^Am by non-modified and oxidized biochar fibers at laboratory-prepared solutions at picomol levels; (b) study the effect of pH, adsorbent mass, ionic strength (I), temperature, and the water type used on the removal efficiency and determine the adsorption efficiency in terms of the *K*_d_ values; and (c) compare the performance of the biochar adsorbent in three distinctly different aqueous environments: seawater, groundwater, and wastewater.

## 2. Results and Discussion

### 2.1. Effect of Contact Time on the ^241^Am Adsorption

**Figure 1:**
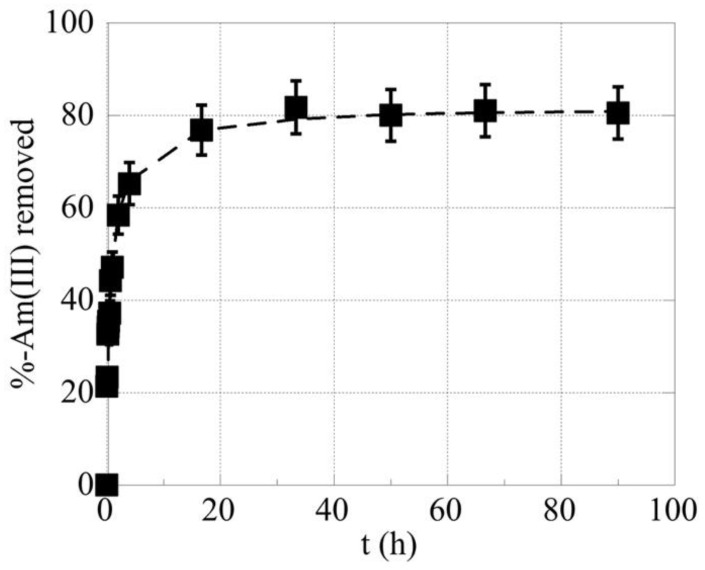
The removal % of Am^3+^ as a function of time. [^241^Am] = 1 × 10^−12^ mol/L, biochar mass = 0.01 g, equilibrium pH = 4, and the ambient conditions.

Adsorption kinetics describes the relationship between the mass transfer of the adsorbate and time in the adsorption process, which is helpful to determine the time needed for the system to reach equilibrium as well as the kinetic parameters associated with the rate of the adsorption process. The time needed to reach equilibrium in the system has been investigated by determining the relative quantity of Am^3+^ adsorbed by the oxidized biochar fibers as a function of contact time. The associated kinetic data are summarized in Figure 1 and reveal that equilibrium was reached after 24 h. Therefore, subsequent experiments have been carried out allowing for 24 h contact time to assure equilibrium. In the sub-picomolar concentration range, equilibrium was reached after 24 h contact time, which is a significantly higher contact time compared to the few hours required at increased radionuclide/metal ion concentrations [7,8,9,24,38]. This is because at very low concentration levels, the diffusion of the radionuclide cations to the biochar surface is the adsorption rate determining step, and the mass transfer phenomena are reduced. Shorter equilibrium times in the range of 30–180 min have been reported in the literature for ^241^Am, however, using much higher Am^3+^ concentrations and highly modified, silica-based adsorbents [38,39]. Furthermore, the kinetic data have been fitted with the pseudo-first-order (PFO) and pseudo-second-order (PSO) kinetic models and the results indicate that the data were better fitted with the PSO (R = 0.999) compared to the PFO (R = 0.952) kinetic model.

### 2.2. Effect of pH on ^241^Am Adsorption

**Figure 2:**
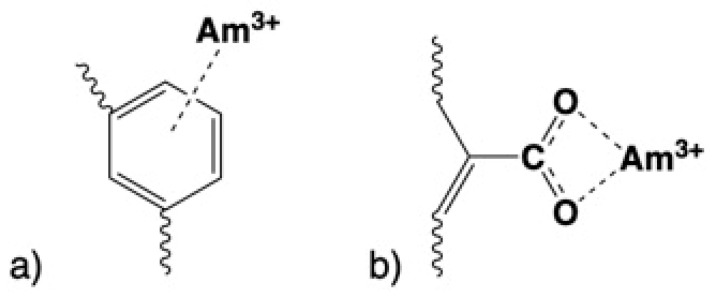
Schematic illustration of the interaction of the Am^3+^ cation with (**a**) the aromatic (π-system) and (**b**) the carboxylic groups on the biochar surface.

The solution pH strongly affects the sorption efficiency (*K*_d_ values), since both the Am^3+^ species distribution in solution and the protonation/dissociation of the surface-active moieties of the biochar materials depend on the proton concentration. The latter applies to the π system–proton interaction and carboxylic dissociation for unmodified and oxidized biochar, respectively [20,21,22,23,24]. A schematic illustration of the interaction of Am^3+^ cations with the aromatic (π-system) and the carboxylic groups on the biochar surface is given in Figure 2.

Depending on the solution pH, Am^3+^ exists in acidic solutions predominantly in the form of the Am^3+^ cation, at near neutral solutions predominantly as Am^3+^, Am(OH)_2_^+^, and Am(CO_3_)^+^, and under alkaline conditions, the stable Am(CO_3_)_2_^−^ is the dominating species [4,5,6,40]. Regarding the biochar surface, at pH = 2, the carboxylic moieties were extensively protonated, because the mean value of the acid dissociation constant, pK_a_, was below 4. At pH 4, a significant number of the carboxylic moieties was deprotonated and only at pH = 7 and 9 did the deprotonated carboxylic groups dominate, and the biochar surface was negatively charged [20,21,22,23].

**Figure 3:**
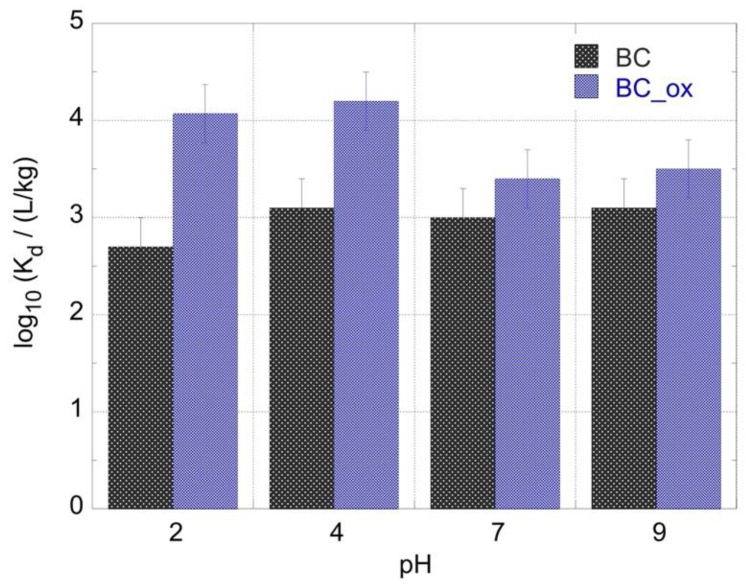
*K*_d_ values for the Am^3+^ adsorption by oxidized biochar fibers as a function of pH. [^241^Am] = 1 × 10^−12^ mol/L, biochar mass = 0.01 g, ambient conditions.

The effect of pH on the Am^3+^ adsorption by biochar fibers has been investigated and the corresponding log_10_*K*_d_ values are summarized in Figure 3. The oxidized biochar fibers (BC_ox) showed a much higher adsorption affinity for Am^3+^ than their non-oxidized counterpart (BC), which may be attributed to the presence of a significantly higher concentration of oxygen-containing moieties (e.g., carboxylic groups) on the BC-ox surface [20,21,22,23,24]. Oxygen-containing moieties (e.g., phenolic, carboxylic groups) are hard Lewis bases and therefore exhibit a higher affinity for hard Lewis acids such as the Am^3+^ cationic species. Hence, the highest *K*_d_ values for BC_ox were observed in the acidic pH region log_10_*K*_d_ (BC_ox) = 4.1 ± 0.1 at pH = 2 and log_10_*K*_d_ (BC_ox) = 4.2 ± 0.2 at pH = 4, and the lowest values (log_10_*K*_d_ (ΒC) = 3.4 ± 0.3 at pH = 7 and log_10_*K*_d_ (ΒC_ox) = 3.5 ± 0.2 at pH = 9. The significantly higher affinity of the oxidized biochar for Am^3+^ in the acidic pH region can be attributed to the predominance of the Am^3+^ cation in the respective pH region and the partially deprotonated, negatively charged surface carboxylic moieties, which strongly attract and complex the Am^3+^ cations, resulting in the formation of inner-sphere complexes [20,21,22,23,24]. These observations agree well with Li et al. (2001) and Zhang et al. (2014), who reported comparable *K*_d_ values at acidic pH [33,40]. In the neutral and alkaline pH regions, the progressive formation of the Am(OH)_2_^+^, Am(CO_3_)^+^, and Am(CO_3_)_2_^−^ species resulted in a decline in the electrostatic attraction and to some extent hindered the surface complex formation. On the other hand, the pristine biochar fibers presented the lowest *K*_d_ value in the strongly acidic pH region (log_10_*K*_d_ (ΒC) = 2.1 ± 0.3 at pH = 2), whereas the values at pH 4, 7, and 9 were comparable to the values obtained by BC_ox. The lower sorption affinity of pristine biochar for Am^3+^ at pH = 2 is related to the increased concentration of protons, which compete with the Am^3+^ cations based on cation–pi interaction between the positively charged species and the aromatic rings of the biochar [20,21,22,23,24]. Generally, the effect of pH on the Am^3+^ adsorption by biochar fibers in the picomolar concentration range is similar to the adsorption of its chemical analogue (Eu^3+^) [39,40,41].

### 2.3. Effect of Ionic Strength on ^241^Am Adsorption

**Figure 4:**
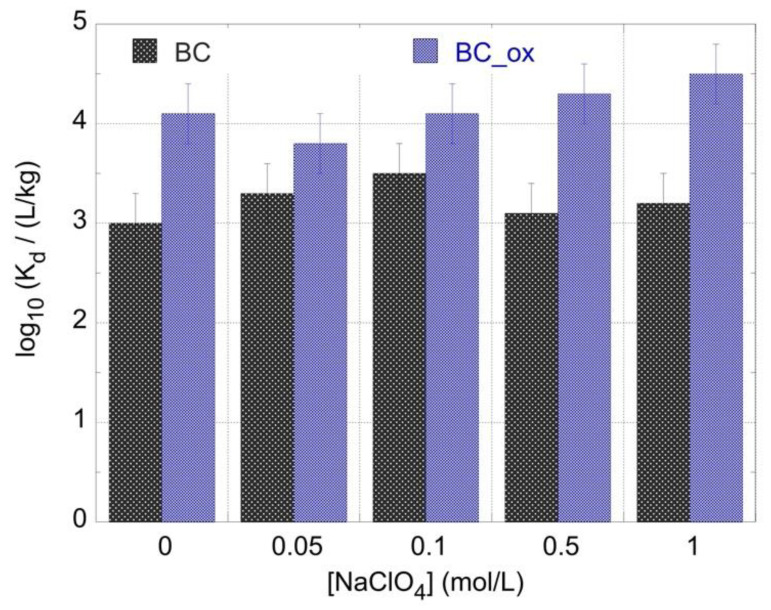
*K*_d_ values for the ^241^Am adsorption by oxidized biochar fibers as a function of ionic strength. pH = 4, [^241^Am] = 1 × 10^−12^ mol/L, biochar mass = 0.01 g, ambient conditions.

At increased metal ion concentrations, spectroscopic characterization (Fourier transform, X-ray photoelectron, and Raman) of the adsorbed species and evaluation of the adsorption mechanism at the molecular level is feasible, however, at ultra-trace levels, this cannot occur. Therefore, the effect of the ionic strength on the adsorption efficiency was investigated to gain insights on the adsorption efficiency and evaluate the type of surface complexes formed (e.g., outer- or inner-sphere complexes). Generally, as the ionic strength of the solution increases, a decline of the adsorption efficiency is observed, characteristic of non-specific, simple electrostatic interactions and the predominance of outer sphere complex formation. However, when high ionic strength does not result in a decline in the adsorption efficiency, the existence of specific interactions and the formation of inner-sphere complexes governs the adsorption process [20,21,22,23,24]. Figure 4 shows that the adsorption efficiency (log_10_*K*_d_ values) was not reduced with increasing ionic strength, assuming specific interactions and the formation of inner-sphere complexes between Am^3+^ and active moieties (e.g., aromatic rings and carboxylate groups) on the biochar surface. This is in agreement with previous studies on the adsorption of trivalent lanthanides by oxidized biochar fibers, which had been performed at increased metal ion concentration (at the mmol/L concentration range) and by means of spectroscopic investigations such as FTIR [41] and XPS [42], which indicated the formation of inner-sphere complexes between the metal ion and the surface-active moieties.

### 2.4. Effect of Temperature on ^241^Am Adsorption

The thermodynamic parameters (Δ*H*° and Δ*S*°) of the Am^3+^ adsorption by the pristine and oxidized biochars were evaluated by determining the associated *K*_d_ values at three different temperatures and plotting ln*K*_d_ versus 1/*T*, according to the van’t Hoff equation:

(1) $$\ln K_{d}=-\frac{\Delta H^{{}^{\circ}}}{RT}+\frac{\Delta S^{{}^{\circ}}}{R}$$

**Figure 5:**
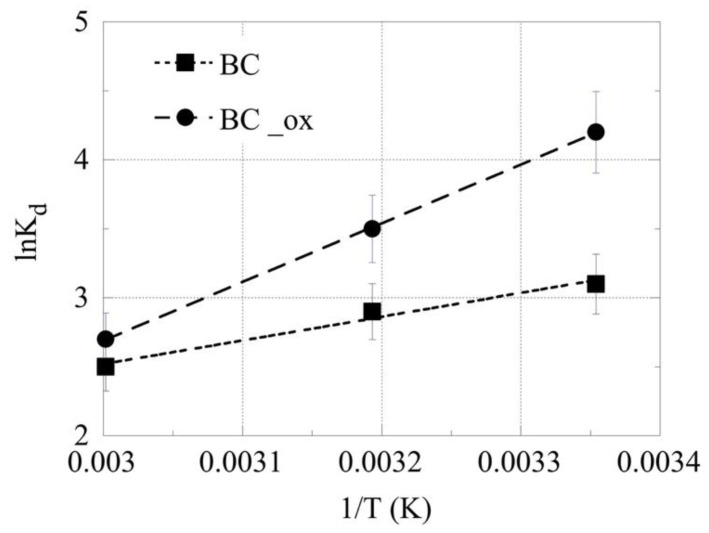
ln*K*_d_ as a function of 1/*T* for the adsorption of Am^3+^ by oxidized biochar fibers at an initial ^241^Am concentration of 1 × 10^−12^ mol/L, biochar mass = 0.01 g, pH = 4, and 3 days of contact time.

The plot of ln*K*_d_ against 1/*T* is shown in Figure 5. The thermodynamic parameters were evaluated by the slope and intercept obtained from the linear regression of the corresponding experimental data as Δ*H*° = −206 kJ mol^−1^ and Δ*S*°= −0.5 J K^−1^ mol^−1^, Δ*H*° = −512 kJ mol^−1^ and Δ*S*°= −1.2 J K^−1^ mol^−1^, for the adsorption of Am^3+^ on the pristine and oxidized biochar, respectively. Furthermore, the Δ*G*° values at 25, 40, and 60 °C for the pristine and oxidized biochar were −56.9 kJ mol^−1^, −49.4 kJ mol^−1^, −39.4.9 kJ mol^−1^ and −154.2 kJ mol^−1^, −136.2 kJ mol^−1^, −112.4.2 kJ mol^−1^, respectively. These values indicate an exothermic adsorption process, in contrast to the observations obtained from the adsorption of U(VI) on the same adsorbents and the same concentration range. The corresponding values for U(VI) were Δ*H*° = 1.5 kJ mol^−1^ and Δ*S*° = 1.6 kJ K^−1^ mol^−1^ [23]. Moreover, these thermodynamic parameters differed from the corresponding parameters obtained from the experiments performed using Sm^3+^ as the Am^3+^ analogue (Δ*H*° = 34 kJ mol^−1^ and Δ*S*° = 193 JK^−1^ mol^−1^) was performed at higher (mmol range) metal ion concentrations [42]. This could be attributed to the fact that at higher metal ion concentrations, the assumption of a large excess of binding site species is not relevant, and different adsorption mechanisms prevail on the biochar surface.

### 2.5. Removal of ^241^Am from Seawater, Groundwater, and Wastewater

**Figure 6:**
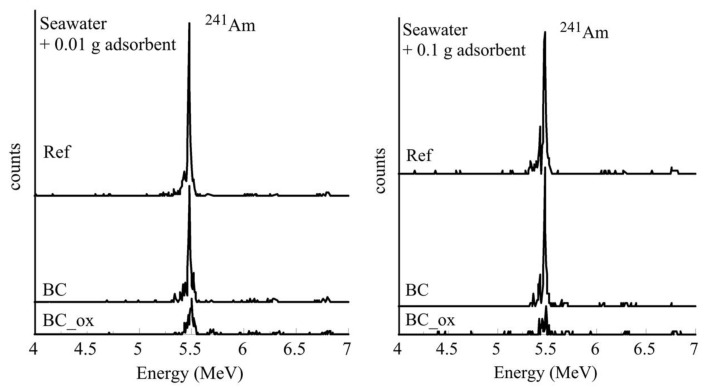
Alpha spectra of ^241^Am contaminated seawater samples prior and after contact with the 0.01 g and 0.1 g biochar fibers prior (BC) and after (BC_ox) oxidation. [^241^Am] = 1 × 10^−12^ mol/L, ambient conditions.

The removal of ^241^Am from the seawater, groundwater, and wastewater from the local wastewater treatment plant was investigated after spiking the respective solutions with ^241^Am and in contact with two different biochar doses of 0.01 and 0.1 g. After a 24 h contact time, the radionuclide concentration in the solution was analyzed by alpha spectroscopy. Figure 5 shows the characteristic alpha-spectra of ^241^Am corresponding to the contaminated seawater samples prior and after treatment with 0.01 and 0.1 g of the pristine and oxidized biochars. The spectra in Figure 6 clearly indicate that the ^241^Am levels declined in the presence of biochar fibers, particularly in the presence of the oxidized counterparts. The latter was attributed to the strong affinity of the carboxylic moieties, which were present on the BC_ox surface [20,21,22,23] for Am^3+^, resulting in the formation of inner-sphere complexes. Figure 6 shows that increasing the amount of biochar resulted in higher removal efficiencies.

**Figure 7:**
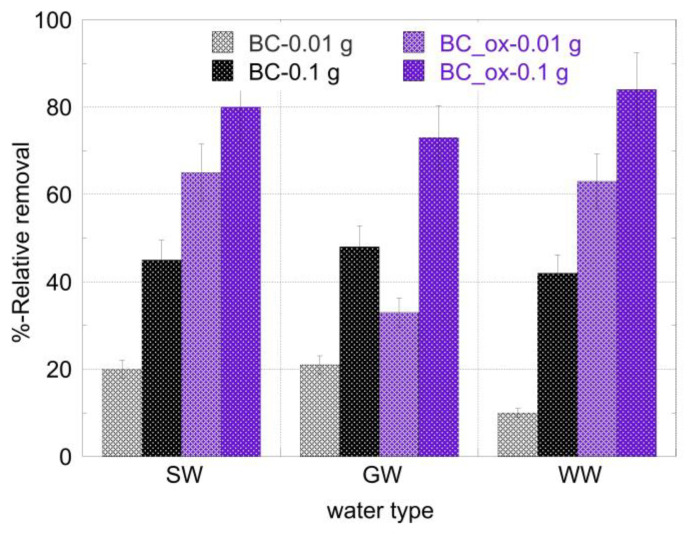
Relative % removal of ^241^Am from the environmental water samples after treatment with 0.01 and 0.1 g of the non-modified (BC) and oxidized (BC_ox) biochar fibers. [^241^Am] = 1 × 10^−12^ mol/L, ambient conditions.

Based on the alpha spectra obtained for the three different aqueous matrices and the two different doses of BC and BC_ox, the relative quantity of Am^3+^ removed from the solution was calculated and the corresponding data are graphically summarized in Figure 7. The oxidized biochar fibers presented the highest removal efficiency (~80%) in all three natural waters. Increasing the biochar dose from 0.01 to 0.1 g resulted in a 10–40% increase in the removal efficiency, depending on the aqueous system. At the highest adsorbent dose of 0.1 g, the removal of ^241^Am by the oxidized biochar was twice that of the pristine sample (~80 compared to ~40%) for the seawater and wastewater. In the case of the groundwater, the efficiency of the oxidized biochar was slightly reduced, but was nevertheless considerably higher compared to the pristine biochar, with 70% removal compared to ~50%, respectively. The slightly decreased performance of the oxidized biochar on the groundwater may be attributed to the elevated concentrations of Ca^2+^ and Fe^3+^ ions, which may compete with Am^3+^ for sites on the adsorbent surface [23,42]. These observations confirm the high adsorption affinity of the oxidized biochar fibers toward Am^3+^ and establish the applicability of the material for the treatment of americium contaminated natural waters and wastewaters.

**Figure 8:**
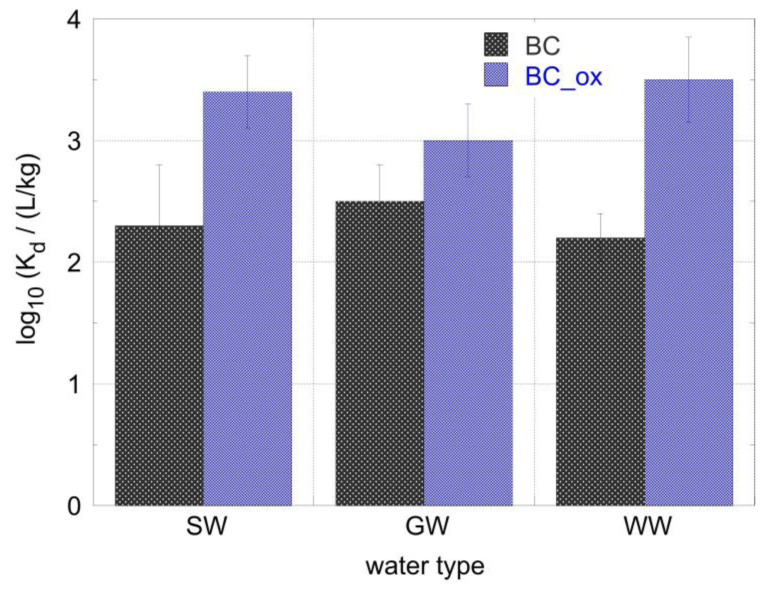
*K*_d_ values corresponding to ^241^Am adsorption from the environmental water samples in contact with the pristine (BC) and oxidized (BC_ox) biochar fibers. [^241^Am] = 1 × 10^−12^ mol/L, ambient conditions.

Furthermore, the alpha spectroscopic data were used to evaluate the *K*_d_ values associated with each biochar sample for the adsorption of Am^3+^ from the aqueous solutions corresponding to three different water systems. The calculated *K*_d_ values are summarized in Figure 8 and were similar (log_10_*K*_d_ ~ 3.5 at pH = 7 and pH = 9) to the corresponding values determined in the laboratory, de-ionized water solutions for the oxidized biochar (BC_ox), and about half a logarithmic unit lower (log_10_*K*_d_ ~ 2.5 at pH = 7 and pH = 9) for the non-oxidized counterpart. The lower *K*_d_ values associated with the seawater samples can be attributed to the presence of competing cations in the natural water system (e.g., Ca^2+^, Fe^3+^), which may be because hard Lewis acids interact and occupy the surface active sites on the biochar surface, resulting in adsorption affinity for the Am^3+^ cations in the respective systems [23,41]. Moreover, in the case of the pristine biochar, even conservative cations (e.g., Na^+^, K^+^) may interact with the biochar surface via cation–π system interactions, significantly affecting the Am^3+^ adsorption by the pristine biochar materials. Nevertheless, the evaluated *K*_d_ values were similar to the mean linear distribution value (log_10_*K*_d_ = 3.7) reported for Am^3+^ in soils [43], indicating the supreme affinity of the oxidized biochar fibers for Am^3+^.

## 3. Materials and Methods

All experiments were carried out in 30 mL polyethylene (PE) screw capped bottles under ambient conditions (23 ± 2 °C). The americium isotope, ^241^Am, was employed in the experiments (^241^Am standard tracer solution, 7.367 kBq/g, North American Scientific Inc., Los Angeles, CA, USA). This standard solution was diluted to prepare reference and test solutions with an initial concentration of 1 mBq/mL. The biochar samples were prepared from the pyrolysis of the vegetable sponge gourd. The preparation of the biochar was performed by carbonization at 650 °C under inert (N_2_) atmosphere for two hours, the oxidation was carried out by treating the biochar with 8 M HNO_3_ for 1 h, and the characterization of the materials by surface and spectroscopic methods has been extensively described elsewhere [20,21,22]. It is worth mentioning that, in contrast to the non-modified biochar fibers, which are characterized by the presence of graphite sheets, and the carboxylic moieties govern the surface charge and chemistry of oxidized biochar fibers. The experiments were performed in laboratory solutions using de-ionized water at different pH regions (pH 2, 4, 7, and 9) and in naturally-occurring aqueous matrices such as groundwater (GW), wastewater (WW), and seawater (SW) samples. The groundwater was sampled from a local well; the wastewater, which corresponds to the effluent of secondary treatment, was obtained from a municipal wastewater treatment plant; and the seawater sample was collected from a coastal area of the island. The pH and main components of the environmental waters, which have been analyzed as described elsewhere [10,11], are summarized in Table 1.

The pH in the laboratory solutions was adjusted using either 0.1 M HCl or 0.1 M NaOH solutions and the pH measurements were carried out using a conventional laboratory pH meter (pH 211 microprocessor pH meter, Hanna Instruments, Woonsocket, RI, USA). The radiometric analysis of ^241^Am was carried out using an alpha-spectrometer (Alpha Analyst Integrated Alpha Spectrometer, Canberra Industries, Fussy, France), as described elsewhere [7,38]. In addition, reference and control samples of the radionuclide were also analyzed by liquid scintillation counting (LSC, Triathler, Hidex, Turku, Finland). Alpha-spectrometric analysis was performed in triplicate and the LSC measurements were carried out in parallel to compare and validate the data obtained from both radiometric methods. The mean values and the associated standard deviations were used for the graphical presentations. The detection limits were determined at 0.05 mBq and 0.03 mBq for the LSC and alpha-spectrometric measurements, respectively.

The adsorption studies were carried out by adding 0.01 or 0.1 g of the biochar to 20 mL of the radionuclide solution at an activity concentration of 25 Bq/L ([^241^Am] = 1 × 10^−12^ mol/L) in 30 mL screw capped PE vials. The effect of contact time was investigated at pH = 4 and under the aforementioned experimental conditions. The effect of pH was studied at pH = 2, 4, 7, and 9, and the effect of ionic strength (I) was investigated using aqueous NaClO_4_ solutions at various concentrations (no electrolyte added, 0.05, 0.1, 0.5, and 1 Μ). The effect of temperature was investigated at 25, 40, and 60 °C, at pH = 4. The biochar-radionuclide solution suspensions were agitated on a shaker (SK-R1807, DLAB, Beijing, China) at an agitation rate of 65 rpm for 24 h, after which the suspension was allowed to rest. For the analysis of ^241^Am, aliquots of 200 μL were obtained from the supernatant to evaluate the radionuclide concentration in solution by alpha-spectrometry or liquid scintillation counting. The radiometric methods were calibrated using standard reference solutions and sources. The sorption efficiency was evaluated using the partition coefficient (*K*_d_). The *K*_d_ applies because of the picomolar ^241^Am concentrations used and the relatively large excess of the available surface binding sites. The following equation describes the partition coefficient:

(2) $$K_{d}=[{\mathrm{Am}}^{3+}{]}_{\mathrm{ads}}/[{\mathrm{Am}}^{3+}{]}_{\mathrm{aq}}\;\left(\frac{L}{\mathrm{kg}}\right)$$

where [Am^3+^]_ads_ is the activity of ^241^Am adsorbed by the biochar fibers (Bq/g) and [Am^3+^]_aq_ (Bq/L) is the ^241^Am activity concentration in solution at equilibrium. The quantity of ^241^Am adsorbed by biochar fibers (dry mass) was calculated from the total ^241^Am activity adsorbed by subtracting the ^241^Am activity adsorbed by the vial walls. The latter was not negligible and had to be taken into account. Therefore, for each separate test solution (also including the effect of pH, temperature, and other parameters), a reference solution under exactly the same conditions (without the biochar material) was prepared, and the activity concentration of americium was determined in parallel. In addition, under the experimental conditions, Am^3+^ is expected to be the predominant oxidation state in solution [1,2,3,4,5,6,7]. Furthermore, the sorption efficiency was expressed as % relative removal and was calculated as follows:

(3) $$\mathrm{relative}\;\%\;\mathrm{removal}=100\times {\left([{\mathrm{Am}}^{3+}\right]}_{R}-{\left[{\mathrm{Am}}^{3+}\right]}_{\mathrm{aq}}/{\left[{\mathrm{Am}}^{3+}\right]}_{R}\;)$$

where [Am^3+^]_R_ is the ^241^Am concentration in the reference solutions.

The experiments were performed in triplicate and the mean values and uncertainties were used for the data evaluation and graphical presentations.

## 4. Conclusions

Given the scarcity of data on treatment methods for ^241^Am-contaminated waters and the established hazard from the emission of alpha particles from its decay, there is a clear need to develop and optimize the adsorption processes. In this work, it was demonstrated that a low-cost, carbonaceous adsorbent is suitable for the treatment of ^241^Am radioactivity in a range of aqueous environments, at very low concentrations. Compared to conventional adsorbents based on silica or other inorganic oxides, biochar has the added advantage of coming from a renewable biomass, thus rendering it more environmentally friendly. Of the two biochar samples tested, it appeared that the oxidized biochar was more efficient due to the higher number of carboxylic moieties. The performance of this sample was maintained at more complex, naturally-occurring waters, a promising observation toward scaling-up of the process. Furthermore, biochar materials, in general, particularly after oxidation, exhibit high physical and chemical stability. In addition, numerous previous investigations on the recoverability of biochar after adsorption have shown that the material retains its increased adsorption efficiency even after five adsorption–desorption cycles.

Since the published results on the treatment of radionuclide-contaminated water and wastewater by biomass-based materials is limited, future work will focus on preparing engineered biochars to further improve the adsorption capacity. Additionally, given the complexity of real wastewater samples from nuclear power stations, the selectivity of such adsorbents for specific residual radionuclides should be investigated.

## Figures and Tables

**Table 1:** The pH values and levels of the main ions identified in the aqueous matrices used in this study.

Parameter (mg/L)	Wastewater	Groundwater	Seawater
pH	8.1	7.8	8.3
K^+^	29	<3	395
Na^+^	nd ^a^	40	10,680
Ca^2+^	87	38	410
Mg^2+^	55	70	1280
Fe^3+^	nd	<35	0.003
Cu^2+^	nd	<50	0.09
Cl^−^	298	54	19,200
HCO_3_^−^	nd	370	140
SO_4_^2−^	111	95	2680

^a^ Not detected.

## Data Availability

Data are contained within the article.
